# Influence of Mulberry Leaves on the Fermentation Characteristics and Nutritional Value of Sugarcane Silage

**DOI:** 10.3390/ani16050819

**Published:** 2026-03-05

**Authors:** Jozivaldo Prudêncio Gomes de Morais, Mariana Campana, Maria Eduarda Pieniz Hamerski, Estefani Capucho, João Gustavo Trofino Carassato, Giovani Vignola Tirloni, Ana Caroline Rossi, Tiago Antonio Del Valle

**Affiliations:** 1 Departamento de Biotecnologia e Produção Vegetal e Animal, Centro de Ciências Agrárias, Universidade Federal de São Carlos, Araras 13604-900, SP, Brazil; jozivaldo@ufscar.br (J.P.G.d.M.); mariana.campana@ufscar.br (M.C.); estefanicapucho2@gmail.com (E.C.); joaocarassato@hotmail.com (J.G.T.C.); giovanitirloni@estudante.ufscar.br (G.V.T.); carolrossi@ufscar.br (A.C.R.); 2 Department of Animal Science, Rural Sciences Center, Federal University of Santa Maria, Santa Maria 97105-900, RS, Brazil; meduardahamerski@gmail.com

**Keywords:** acidification kinetics, ensiling, feed additive, moisture adsorbents, *Morus nigra*

## Abstract

Sugarcane ensiling is often compromised by low dry matter (DM) and high soluble carbohydrate content, which promote undesirable alcoholic fermentation and substantial nutrient losses. This study evaluated the inclusion of dehydrated mulberry leaves (*Morus nigra*) as a natural additive to modulate the fermentative profile and improve the quality of sugarcane silage. Treatments consisted of sugarcane silage without additives (CON) or with mulberry inclusion (MUL; 24 g/kg as-fed). During ensiling, CON silages acidified faster, whereas MUL silages maintained higher pH values and greater preservation of soluble solids. At silo opening (60 d), MUL silages exhibited lower ammonia nitrogen and a 59.1% reduction in total DM losses, primarily driven by reduced effluent production. Lactic acid concentration remained unaffected by treatments. Mulberry inclusion significantly enhanced the nutritive value by increasing DM and crude protein contents while reducing fiber fractions. Consequently, in vitro degradation of DM and neutral detergent fiber (NDF) increased by 27.8% and 72.6%, respectively. Upon aerobic exposure, MUL silages showed altered pH and soluble carbohydrate dynamics compared to CON. In conclusion, including mulberry leaves is an effective and sustainable strategy to mitigate fermentative losses, as well as improve the nutritive value of sugarcane silage.

## Simple Summary

Sugarcane is an important feed for cattle in tropical regions, but its daily harvest is labor-intensive. While ensiling is a solution for year-round storage, sugarcane’s high sugar content often causes excessive fermentation losses and poor nutritional quality. This study evaluated mulberry leaves as a sustainable, farm-grown additive to solve these issues. Our results demonstrate that adding mulberry leaves significantly reduces nutrient losses during fermentation and improves the protein content and digestibility of the silage. By using a plant easily cultivated on farm, this research provides producers with a cost-effective and natural strategy to optimize animal nutrition. These findings contribute to the research community by offering a practical approach to minimize feed waste and promote more resilient and sustainable livestock production systems.

## 1. Introduction

Sugarcane serves as a forage source in tropical ruminant production due to its high biomass yield and cost-effectiveness [1]. However, the logistical constraints of daily harvesting and wildfire risks during the dry season necessitate ensiling as a conservation strategy [2,3]. While ensiling ensures year-round feed availability, sugarcane’s low dry matter (DM) and high soluble carbohydrate content trigger a critical preservation challenge [4].

Under these conditions, yeasts metabolize sucrose and glucose into ethanol and CO_2_ [5]. This alcoholic fermentation not only causes substantial DM and energy losses but also compromises aerobic stability upon silo opening [6,7,8]. To mitigate these losses, researchers have tested chemical and microbial additives [9,10,11,12]; however, while urea or calcium oxide can inhibit yeasts, they often fail to improve the overall nutritional profile or are restricted by cost and handling risks.

Consequently, there is an urgent demand for sustainable, “on-farm” natural additives that simultaneously modulate fermentation and enhance silage nutritive value. Mulberry leaves (*Morus nigra*) emerge as a promising candidate due to their high crude protein content, low fiber fraction, and bioactive antioxidant compounds [13,14,15]. Beyond their nutritional benefits, dehydrated mulberry leaves may act as moisture adsorbents, increasing the DM of the ensiled mass to create an environment less conducive to spoilage [16].

Despite the potential of mulberry in other forage systems, its specific impact on sugarcane silage remains unexplored. Therefore, we hypothesized that including dehydrated mulberry leaves would shift the fermentative pattern, reducing ethanol-related losses and improving the nutritive value and aerobic stability of the silage. Thus, the objective of this study was to evaluate the effects of including dehydrated mulberry leaves on the fermentation profile, losses, chemical composition, in vitro degradation, and aerobic stability of sugarcane silage.

## 2. Materials and Methods

The experiment was conducted at the Centro de Ciências Agrárias (CCA) of the Universidade Federal de São Carlos, located in the municipality of Araras, São Paulo State, Brazil, from June to September 2023.

### 2.1. Treatments and Experimental Design

The experiment followed a randomized complete block design, with sugarcane cultivars (RB085436, RB085452, RB085446, and RB085472, Ridesa Ufscar, Araras, Brazil) serving as blocks (*n* = 4). Treatments consisted of: (1) Control (CON): sugarcane silage without additives; and (2) Mulberry (MUL): sugarcane silage with 24 g/kg (as-fed) or 86 g/kg (DM basis) of dehydrated black mulberry leaves. Experimental units (*n* = 8) were defined as treatment × block interaction effect. A total of 32 experimental units were used (8 for CON and 24 for MUL) to correctly evaluate the variables.

### 2.2. Ensiling, Sampling, and Aerobic Stability

Sugarcane was harvested manually and processed in a stationary chopper to a particle size of 1.5–2.0 cm. Black mulberry leaves (*Morus nigra*) were similarly chopped to ensure uniform mixing. Representative samples of each sugarcane cultivar and the mulberry leaves were collected prior to ensiling to determine particle size [17] and initial chemical composition (Table 1). Forage was manually mixed according to treatment proportions and packed into two types of experimental units. For the final evaluation (60 days), 32 PVC silos (30 cm diameter × 30 cm height) were filled to a target density of 650 kg fresh matter/m^3^. These units were equipped with a 5 kg layer of dry sand at the bottom for effluent collection. To monitor fermentation kinetics, a total of 192 vacuum-sealed plastic mini-silos (1 L each) were prepared. All silos were hermetically sealed and stored at room temperature (25 ± 2 °C). A destructive sampling design was employed, in which a dedicated set of 32 silos was opened at each sampling interval (12, 24, 48, 72, 120, and 168 h post-ensiling). Upon opening, the entire contents of each mini-silo were homogenized, and representative samples were collected for pH and soluble solids (Brix) analysis. Once sampled, these mini-silos were discarded and not reused for subsequent time points.

The PVC silos were weighed both at ensiling and opening to quantify fermentative losses. Upon opening, the silage was thoroughly homogenized, and a 500 g aliquot was subjected to a hydraulic press to extract the silage juice. The pH of the juice was immediately measured using a benchtop pH meter (PH2500; Marte Científica, Santa Rita do Sapucaí, Brazil). Soluble solids (Brix) were measured by refractometry [18] (method 990.35), representing the compounds dissolved in the plant juice, such as sugars, proteins, and minerals, which serve as substrates for microbial fermentation of the silage. Sub-samples of the fresh silage (unpressed) and of the juice extracted from the pressed portion were frozen and maintained at −20 °C until chemical analysis. For the aerobic stability test, 3 kg samples were placed in buckets (without compaction, simulating the feed-out phase) and kept uncovered in a climate-controlled room at 24 °C for 168 h. This duration was chosen to rigorously evaluate the impact of mulberry inclusion under conditions that favor rapid spoilage, as observed in tropical environments. Every 24 h, 15 g samples were collected to monitor pH and soluble solids content as described previously.

### 2.3. Chemical Analysis and In Vitro Assay

The silage fluid was thawed at room temperature, centrifuged (500× *g* for 15 min), and the supernatant was analyzed for fermentation products. Ammoniacal nitrogen was determined using the Kjeldahl method [18] (method 984.13), omitting the digestion step. Lactic acid was analyzed using a spectrophotometric method [19].

Samples frozen for chemical composition analysis were dried in a forced-air oven at 65 °C for 72 h and then ground in knife mills with 1 mm and 2 mm sieves to allow evaluation of chemical composition and in vitro degradation. Fresh sugarcane, mulberry, and silage samples were analyzed for dry matter [18] (DM; method 950.15), organic matter [18] (OM; calculated as 100 − ash, method 942.05), ether extract [18] (EE; method 920.39), and crude protein (CP; N × 6.25, method 984.13). The neutral detergent fiber (NDF) and acid detergent fiber (ADF) fractions were determined according to [20], using α-amylase and without the addition of sodium sulfite.

For the evaluation of in vitro ruminal degradation of DM and NDF, ruminal fluid was collected from two Holstein heifers (500 kg body weight) maintained on *Megathyrsus maximus* pasture without concentrate supplementation, via rumen cannula, and stored in anaerobic bottles heated to 39 °C until transported to the laboratory. The ruminal fluid was filtered through two layers of cheesecloth and mixed with McDougall’s [21] buffer (1948) at a ratio of 0.4 L of ruminal fluid (20%) to 1.6 L of buffer (80%), and then gassed with CO_2_.

The samples, processed to a 2 mm particle size [22] and placed in non-woven fabric (NWF) bags measuring 5 × 5 cm with a density of 100 g/m^2^ [23], were incubated in a Daisy-type incubator (Ankom Technology, Macedon, NY, USA) at 39 °C for 48 h. The sample weight was adjusted to less than 20 mg DM/cm^2^ [24]. After incubation, the bags were washed under running water to remove soluble residues, then dried at 55 °C for 72 h and at 105 °C for 2 h for the determination of indigestible DM [25]. Subsequently, the samples were analyzed for NDF content, allowing for the calculation of the in vitro degradation of DM and NDF.

### 2.4. Calculations and Statistical Analysis

Fermentation losses and dry matter recovery (DMR) were calculated according to Jobim et al. [26]. Gas losses (GL) were determined by the difference between the full silo weight at ensiling and at opening:

$$GL\left(\frac{g}{kg}DM\right)\;=\;\frac{WSWE(g)\;-\;WSWO(g)}{EDM\;(kg)}$$

where WSWE is the total silo weight after ensiling, WSWO is the total silo weight at opening, and EDM is the ensiled dry matter.

Effluent losses (EL) were calculated using the weight variation of the empty silo containing the sand layer:

$$EL\left(\frac{g}{kg}DM\right)\;=\;\frac{ESWE(g)\;-\;ESWO(g)}{EDM\;(kg)}$$

where ESWE is the empty silo weight at ensiling, and ESWO is the empty silo weight at opening. Total fermentation losses (TFL, g/kg DM) were calculated as the sum of GL and EL.

Dry matter recovery (DMR, g/kg) was determined by the ratio of dry matter at opening to ensiled dry matter:

$$DMR\left(\frac{g}{kg}\right)\;=\;\frac{ODM\;(g)}{EDM\;(kg)}$$

The non-fiber carbohydrate (NFC) content was estimated as:

$$NFC\;\left(\frac{g}{kg}DM\right)\;=\;1000\;-\;(Ash\;+\;CP\;+\;EE\;+\;NDF)$$

where CP is crude protein, EE is ether extract, and NDF is neutral detergent fiber, all expressed in g/kg DM.

Data were analyzed using the PROC MIXED procedure of SAS (version 9.4; SAS Institute Inc., Cary, NC, USA). The variables measured at a single time point (chemical composition, fermentation losses, and in vitro assays) were analyzed according to the following model:

Yijk = µ + Mi + bj:i + eijk

where

Yijk is the observed value;

µ is the overall mean;

Mi is the fixed effect of mulberry inclusion (i = 0, 1);

bj:i is the random effect of block (j = 1 to 4);

eijk is the random residual error, assumed N (0, σ2e).

Variables measured over time (pH and soluble solids during fermentation and aerobic exposure) were analyzed as repeated measures using the following model:

Yijkl = µ + Mi + bj:i + ωijk + Tl + M×Til + eijkl

where

Tl is the fixed effect of evaluation time;

ωijk is the random error associated with the experimental silo;

M×Til is the fixed effect of the interaction between mulberry and time;

eijkl is the random residual error, assumed NRM (0,R).

The *R* matrix represents the variance-covariance structure. Multiple structures (e.g., Compound Symmetry, Autoregressive, and Unstructured) were tested, and the best fit was selected based on the Bayesian methods (BIC). Significant effects were considered at *p* ≤ 0.05, while *p*-values between 0.05 and 0.10 were discussed as tendencies.

## 3. Results

### 3.1. Silage Fermentation Profile

**Figure 1:**
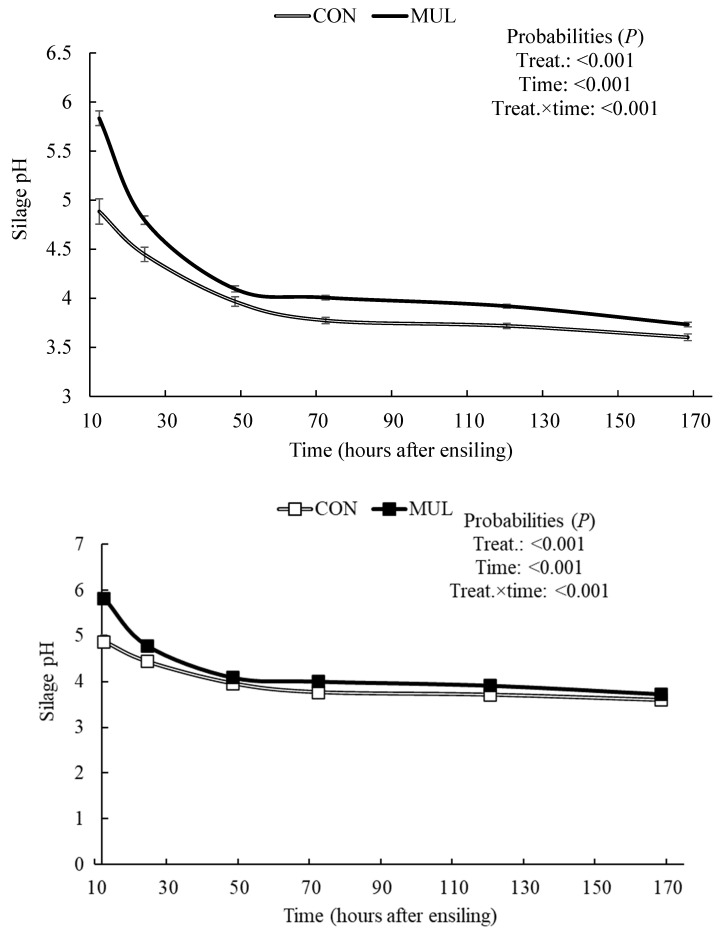
Changes in pH of sugarcane during the early stages of ensiling with or without the inclusion of dehydrated mulberry leaves.

**Figure 2:**
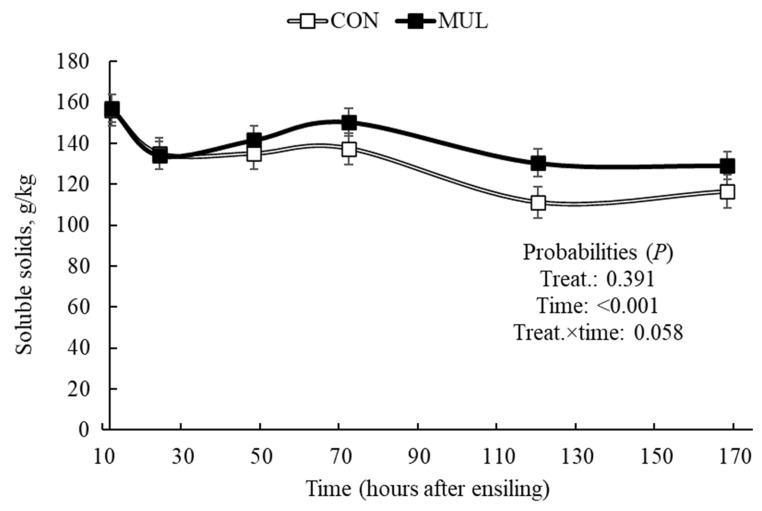
Soluble solids concentration in sugarcane during the early stages of ensiling with or without the inclusion of dehydrated mulberry leaves.

Treatment and fermentation time exerted a combined effect (*p* < 0.001) on silage pH (Figure 1). Although both treatments underwent acidification over time (*p* < 0.001), CON silages maintained a lower pH (*p* ≤ 0.05) during the initial fermentation stages. Consequently, the addition of MUL resulted in a higher final pH (*p* = 0.001) of the silage, with values of 3.51 for MUL and 3.30 for CON (Table 2). In general, treatments did not affect (*p* = 0.391) the soluble solids content of the silage (Figure 2). However, a trend toward a treatment and time interaction effect was observed (*p* = 0.058). Soluble carbohydrate content decreased in both treatments (*p* < 0.001). This reduction was more pronounced in CON silos compared to MUL silos, resulting in a treatment effect (*p* ≤ 0.05) 144 h after ensiling. Consequently, MUL silos showed higher soluble solids values (134 g/kg) compared to CON silos (105 g/kg) at the time of silo opening.

In addition, mulberry inclusion during the sugarcane ensiling reduced NH_3_-N by 41.3% (*p* = 0.012) without affecting (*p* = 0.685) lactic acid concentration (Table 2).

### 3.2. Fermentation Losses

Mulberry addition decreased (*p* < 0.001) silage effluent losses and tended to decrease (*p* = 0.068) gas losses (Table 2). Consequently, MUL showed a 59.1% reduction in total losses compared to CON. However, treatments did not affect dry matter recovery (*p* = 0.340), which averaged 863 g/kg.

### 3.3. Silage Chemical Composition and In Vitro Degradation

Mulberry inclusion increased (*p* ≤ 0.026) silage DM and CP, while reducing (*p* ≤ 0.010) OM, NDF, and ADF content (Table 3). Additionally, MUL tended to increase (*p* = 0.067) silage NFC compared to CON, with no effect (*p* = 0.809) on EE content. Therefore, mulberry enhanced (*p* ≤ 0.031) by 27.8 and 72.6% in vitro degradation of DM and NDF, respectively.

### 3.4. Aerobic Stability Assay

**Figure 3:**
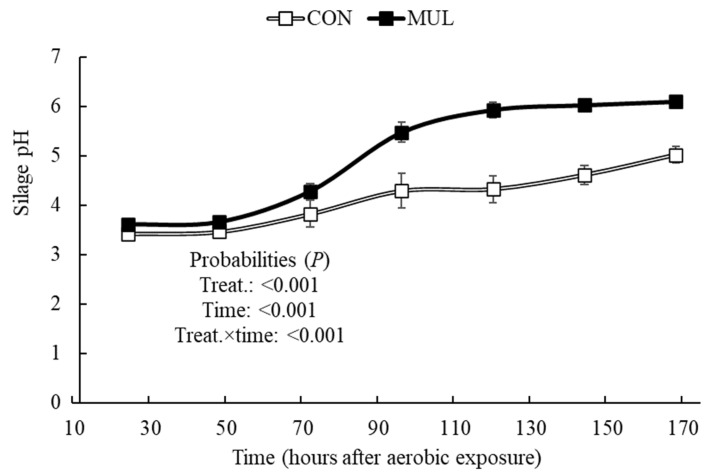
pH of sugarcane silages with or without the inclusion of dehydrated mulberry leaves during aerobic exposure.

**Figure 4:**
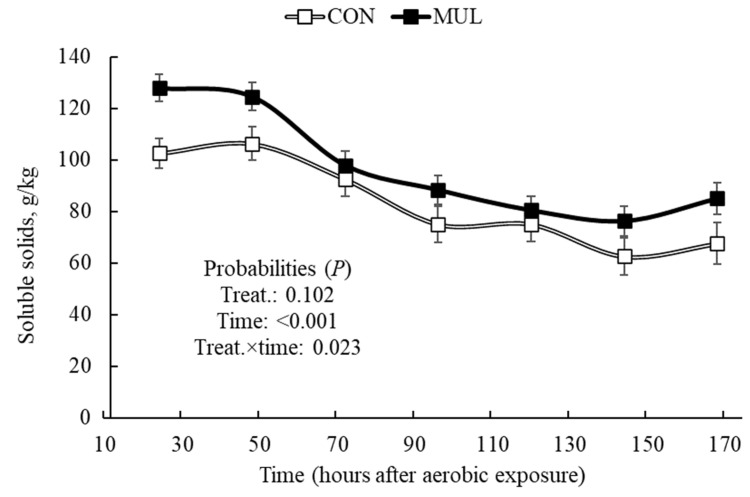
Soluble solids concentration in sugarcane silages with or without the inclusion of dehydrated mulberry leaves during aerobic exposure.

There was an MUL and time interaction effect (*p* < 0.001) on silage pH and soluble carbohydrate content after aerobic exposure (Figure 3 and Figure 4). Compared to CON, MUL silages showed a sharper pH increase over time, especially during prolonged aerobic exposure (Figure 3). Consequently, differences in soluble carbohydrates between treatments disappeared (*p* > 0.05) as exposure time progressed (Figure 4).

## 4. Discussion

This study hypothesized that incorporating dehydrated mulberry leaves into sugarcane silage would attenuate excessive pH drops, mitigate fermentative losses, and enhance both nutritive value and aerobic stability. Our findings largely support this hypothesis, as mulberry inclusion successfully increased silage pH, reduced effluent and total dry matter losses, and improved the overall nutritional profile. However, contrary to our expectations regarding aerobic stability, the MUL treatment exhibited a more rapid pH increase and a faster depletion of soluble solids upon air exposure.

Mulberry inclusion increased the final silage pH and attenuated the initial pH drop, reflecting a distinct acidification pattern compared to the control. The final pH in silages results from the balance between lactic acid synthesis and the forage’s buffering capacity [27]. While pure sugarcane typically exhibits low buffering capacity because of its low protein content, a factor that favors abrupt pH declines [5], mulberry leaves provide higher protein and mineral concentrations that enhance the buffering power [15,28]. The significant time × treatment interaction indicates that mulberry promoted a more controlled acidification process, which potentially prevents excessive hydrolysis. Thus, the inclusion of mulberry raised the final silage pH to 3.51, compared to 3.3 in the CON, remaining closer to the ideal range of 3.8 to 4.2 [5].

Silages containing dehydrated mulberry leaves maintained higher Brix values after 144 h of ensiling and at the final opening compared to CON, despite the overall decrease in soluble solids over time. Soluble solids, primarily sucrose, glucose, and fructose, serve as the essential substrates for microbial fermentation in sugarcane and typically lead to lactic acid production [29]. However, excessive sugar concentrations often trigger yeast proliferation and alcoholic fermentation, which increases ethanol synthesis and dry matter losses [30]. The greater sugar preservation observed in MUL silages suggests that mulberry inclusion successfully modulated carbohydrate consumption, likely by inhibiting inefficient fermentative pathways and preserving the energy density of the forage.

Lactic acid concentrations did not differ between treatments despite the observed variations in sugar preservation. Lactic acid serves as the primary agent in silage conservation because of its high acidifying capacity, which is approximately 10 to 12 times greater than that of other organic acids like acetic or propionic acid [31]. The stable lactic acid levels found in this study suggest that mulberry inclusion did not compromise the homofermentative pathway but rather contributed to more efficient energy preservation by maintaining adequate acidification without excessive sugar depletion. On the other hand, MUL decreased N-NH_3_ concentration compared to CON. The N-NH_3_ levels reflect intense protein degradation, where plant enzymes hydrolyze proteins into peptides and amino acids, followed by microbial conversion into amides, amines, and ammonia [32]. This proteolytic process, often associated with high-moisture silages and clostridial activity, leads to substantial nitrogen losses and reduces the overall nutritive value [29]. The lower N-NH_3_ release in MUL silages indicates superior nitrogen preservation, which likely resulted from the higher DM content of the mulberry leaves.

Mulberry inclusion markedly reduced effluent and total fermentative losses in sugarcane silage. This improvement directly relates to the 6.2% increase in DM content at ensiling (280 vs. 264 g/kg) provided by the dehydrated mulberry leaves. Adjuvants that promote even moderate increases in DM effectively mitigate losses in sugarcane silages by reducing effluent runoff and improving fermentative stability. Similar benefits occurred with the inclusion of forage peanut [33], pigeon pea [34], and rice bran [35], where higher initial DM content consistently reduced gas and effluent production while increasing nutrient recovery. However, MUL inclusion did not significantly affect DM recovery. This phenomenon occurs because effluent losses in sugarcane silages consist predominantly of water (moisture) rather than DM. Therefore, while reducing effluent production is critical for environmental and silos-management purposes, its impact on the final proportion of recovered DM may be negligible, explaining the lack of statistical difference in DMR between treatments.

Beyond the physical effect of moisture adsorption, the reduction in total losses reflects a shift in microbial efficiency. Fermentative losses, primarily driven by CO_2_ production, depend on the predominant microbial species and substrate availability [29,36]). While sugarcane silage typically harbors high yeast populations that convert glucose into ethanol and CO_2_ [5,37]), MUL inclusion reduced gas production. This effect likely stems from the bioactive compounds in mulberry, such as phenolic acids and flavonoids, which possess documented antifungal and antioxidant properties [38]. These compounds potentially inhibited yeast activity, thereby suppressing alcoholic fermentation and undesirable gas production [39]. Consequently, mulberry inclusion optimizes sugarcane silage through an integrated mechanism that combines increased DM content with biological modulation of the fermentative process.

Mulberry inclusion significantly improved the chemical composition of sugarcane silage by increasing DM and CP contents while reducing fiber fractions. The enhanced DM degradation observed in MUL silages stems from both the improved chemical profile and superior preservation of cellular constituents. In typical sugarcane silages, the conversion of sucrose into ethanol reduces DM recovery and proportionally increases the cell wall fraction, which impairs overall digestibility [7,40]. However, the greater sugar preservation and lower intrinsic fiber content of mulberry leaves [15] effectively counteracted this effect, resulting in a more digestible forage.

The NDF degradation of the CON-silage was notably low. While factors such as harvest maturity and genotype were not specifically evaluated in this study, they likely contributed to these findings. For instance, Carvalho et al. [41] reported that advanced maturity in sugarcane increases lignin deposition, directly impairing fiber digestibility. Furthermore, intrinsic genetic characteristics can significantly influence degradability profiles in sugarcane silages [42]. We acknowledge that the use of a single incubation time point (48 h) to assess degradability is a limitation of this study, as it does not capture the complete fermentation kinetics or microbial dynamics. Consequently, the low degradation observed in the CON group highlights the inherent limitations of sugarcane as a sole forage source and underscores the importance of strategies, such as the inclusion of mulberry, to enhance the nutritional value and ruminal degradation of these silages. Future research utilizing multi-point kinetic models and in vivo trials will be essential to further elucidate the mechanistic effects of mulberry inclusion on ruminal efficiency.

Mulberry silages exhibited lower aerobic stability compared to CON, characterized by a faster pH rise and an intensified depletion of soluble solids over the exposure period. Increases in pH during aerobic exposure reflect accelerated microbial activity, which directly compromises silage stability [43,44]. Although MUL silages maintained higher Brix values at opening, these residual soluble solids were rapidly consumed by yeasts and aerobic bacteria. This process triggers the oxidation of fermentable carbohydrates, leading to heat production and a subsequent rise in pH [36,45]. This reduced stability stems from the greater availability of both energy and nitrogen substrates provided by mulberry leaves. The higher CP content and lower initial N-NH_3_ concentration in MUL silages provided a rich environment for aerobic microorganisms at silo opening. Similarly, silages enriched with molasses often show reduced stability due to the high soluble carbohydrate content available for spoilage organisms [46]. These findings indicate that while mulberry leaves optimize the fermentation phase, the resulting nutrient-dense silage requires careful management after silo opening to prevent rapid spoilage.

## 5. Conclusions

The inclusion of dehydrated mulberry leaves in sugarcane silage enhances fermentation by increasing final pH, reducing dry matter losses, and improving chemical composition, with higher DM and CP and lower fiber fractions. Despite lower aerobic stability, mulberry leaves represent an effective and sustainable additive, providing a renewable feed source for ruminants and contributing to the nutritive preservation of the silage.

## Figures and Tables

**Table 1:** Chemical composition and particle size of fresh sugarcane (*n* = 4; mean ± SD) and mulberry leaves (single sample).

Item	Sugarcane	Mulberry
Chemical composition, g/kg dry matter, unless stated		
Dry matter, g/kg fresh matter	264 ± 7.7	944
Organic matter	982 ± 1.9	875
Neutral detergent fiber	417 ± 24.4	409
Acid detergent fiber	227 ± 19.6	143
Non-fiber carbohydrate	532 ± 5.6	292
Ether extract	12.8 ± 2.03	30.2
Crude protein	21.3 ± 2.2	144
Particle size fresh sugarcane ^1^, g/kg		
>19 mm	87.9 ± 22.53	7.54
8 to 19 mm	731 ± 41.7	304
4 to 8 mm	87.5 ± 14.7	79.6
<4 mm	93.3 ± 25.2	609

^1^ Maulfair et al. [17].

**Table 2:** Fermentation profile and losses of sugarcane silage with or without the inclusion of dehydrated mulberry leaves.

Item	Treatments ^1^		*p*-Value ^2^
	CON	MUL	
Fermentation profile			
Silage pH	3.30 ± 0.025	3.51 ± 0.022	0.001
Soluble solids (BRIX), g/kg	105 ± 6.6	134 ± 6.0	0.016
Ammonia-N, g/kg N	109 ± 9.2	64.0 ± 8.56	0.012
Lactic acid, g/kg DM	51.3 ± 4.00	49.0 ± 3.68	0.685
Fermentation losses, g/kg as fed			
Effluent	59.2 ± 4.70	13.8 ± 4.34	<0.001
Fermentation losses, g/kg DM ^3^			
Effluent	238 ± 18.5	52 ± 17.0	<0.001
Gas losses, g/kg DM ^3^	134 ± 10.9	100 ± 10.7	0.068
Total losses, g/kg DM ^3^	372 ± 28.2	152 ± 27.2	0.002
Dry matter recovery, g/kg	843 ± 27.8	883 ± 27.0	0.340

Data are presented as means ± SEM. ^1^ Treatments: CONT: sugarcane silage without the inclusion of dehydrated mulberry leaves; MUL: sugarcane silage with the inclusion of dehydrated mulberry leaves. ^2^ *p*: probability of treatment effect. ^3^ DM: dry matter.

**Table 3:** Chemical composition and in vitro degradation of sugarcane silage with or without the inclusion of dehydrated mulberry leaves.

Item	Treatments ^1^		*p*-Value ^2^
	CON	MUL	
Chemical composition, g/kg dry matter, unless stated			
Dry matter, g/kg as-fed	205 ± 5.7	228 ± 5.5	0.026
Organic matter	974 ± 1.5	963 ± 1.2	0.001
Neutral detergent fiber	608 ± 16.4	516 ± 15.9	0.007
Acid detergent fiber	339 ± 10.8	283 ± 10.6	0.010
Non-fiber carbohydrates	355 ± 15.0	399 ± 12.2	0.067
Ether extract	18.0 ± 3.90	19.1 ± 2.25	0.809
Crude protein	28.4 ± 2.26	46.1 ± 2.02	0.001
In vitro degradation ^3^, g/kg			
Dry matter	474 ± 27.0	606 ± 23.2	0.008
Neutral detergent fiber	135 ± 33.6	233 ± 21.5	0.031

Data are presented as means ± SEM. ^1^ CONT: sugarcane silage without dehydrated mulberry leaves; MUL: sugarcane silage with dehydrated mulberry leaves. ^2^ *p*: probability of treatment effect. ^3^ 48 h in vitro assay.

## Data Availability

The data supporting the findings of this study are available from the corresponding author upon request.
